# A Hybrid Semi-Supervised Anomaly Detection Model for High-Dimensional Data

**DOI:** 10.1155/2017/8501683

**Published:** 2017-11-15

**Authors:** Hongchao Song, Zhuqing Jiang, Aidong Men, Bo Yang

**Affiliations:** Information and Telecommunication Engineering College, Beijing University of Posts and Telecommunications, Beijing, China

## Abstract

Anomaly detection, which aims to identify observations that deviate from a nominal sample, is a challenging task for high-dimensional data. Traditional distance-based anomaly detection methods compute the neighborhood distance between each observation and suffer from the curse of dimensionality in high-dimensional space; for example, the distances between any pair of samples are similar and each sample may perform like an outlier. In this paper, we propose a hybrid semi-supervised anomaly detection model for high-dimensional data that consists of two parts: a deep autoencoder (DAE) and an ensemble *k*-nearest neighbor graphs- (*K*-NNG-) based anomaly detector. Benefiting from the ability of nonlinear mapping, the DAE is first trained to learn the intrinsic features of a high-dimensional dataset to represent the high-dimensional data in a more compact subspace. Several nonparametric KNN-based anomaly detectors are then built from different subsets that are randomly sampled from the whole dataset. The final prediction is made by all the anomaly detectors. The performance of the proposed method is evaluated on several real-life datasets, and the results confirm that the proposed hybrid model improves the detection accuracy and reduces the computational complexity.

## 1. Introduction

Anomalies are known as outliers [1], exceptions [2], aberrations, and surprises [3] in different application domains. Anomaly detection is the identification of samples that do not conform to expected behaviour. In reality, normal samples usually have similar distributions, whereas abnormal samples have different distributions. Anomaly detection has been applied in many fields, including fraud detection [4], intrusion detection [5], and healthcare [6]. Anomaly detection can be generalized as constructing a model from the given training data and predicting the status of unknown data. Various algorithms have been proposed and can be grouped into three classes based on the characteristics of the training data [3]:

- Supervised approaches: both normal and anomalous samples exist in the training dataset, and they are used together to train the detection model. The trained model identifies the test samples as normal or anomalous.
- Semi-supervised approaches: only normal samples are available in the training set; that is, the user cannot obtain information about anomalies. Unknown samples are classified as outliers when their behaviour is far from that of the known normal samples.
- Unsupervised approaches: the class information of all samples in the training data is unknown to the researchers; that is, the samples in the training set may contain both normal and anomalous samples, but the classification of each sample is unknown.

A large amount of labelled training data is required by supervised approaches, and the collection of both positive and negative samples is difficult and time consuming. Furthermore, the detection of new outlier patterns with a model trained on known outliers is challenging. Unsupervised approaches do not require label information for the training data but often suffer from high false alarm rates and low detection rates [7]. In many applications, normal samples are easy to obtain, whereas anomalous samples are expensive to gather; thus, we focus on semi-supervised anomaly detection.

Most of the current anomaly detection approaches are designed for low-dimensional datasets and face challenges as the dimensions increase. Direct application of these approaches to high-dimensional datasets may produce bad results [8]. One widely used method to address this challenge is mapping high-dimensional data into lower-dimensional subspace and processing the new data with conventional detection algorithms. Various dimension-reduction approaches have been proposed, such as feature bagging [9], principle component analysis [10], genetic algorithm [11], linear discriminant analysis [12], and machine learning [13, 14].

The deep belief network (DBN), which consists of layer-stacked restricted Boltzmann machines (RBMs), has been proposed as a multiclass classifier and dimension-reduction tool [15]. Several advantages of DBNs have been identified [13, 16]: they are parametric models whose training time scales linearly with the number of records; they are nonlinear mapping methods that may extract the internal correlations among dimensions; and they can be trained with unlabelled data to represent complex and high-dimensional data in a lower-dimensional subspace. Here, we adopt DBNs as a dimension-reduction tool.

Parameter tuning is another challenging task for parametric semi-supervised anomaly detection. The conventional criteria for measuring the performance of a trained model cannot be used owing to the lack of measurements for outliers. Moreover, the bias between false alarm and false acceptance is difficult to control. Some researchers generated artificial outliers in the validation set to tune the parameters of trained models [3, 13, 17], but artificial outliers may not reflect the distribution of real outliers. Reference [18] proposed a nonparametric adaptive detection algorithm that estimated an anomaly score for each query sample via a nearest neighbor graph. The query sample was classified as anomalous when the score fell below the desired false alarm level. However, the nearest neighbor graph was calculated in full space, so it might suffer from the curse of dimensionality in high-dimensional data.

In this paper, we propose a semi-supervised anomaly detection model for high-dimensional data that consists of two components: a deep autoencoder (DAE) and an ensemble *K*-nearest neighbor graphs- (*K*-NNG-) based anomaly detector. The DAE is trained in unsupervised mode and is used to map high-dimensional data into a feature space with lower dimensionality. This process solves the curse of dimensionality that exists in nearest neighbor calculations. Several anomaly detectors are then built from randomly sampled subsets. This process greatly reduces the computational cost of calculating the nearest neighbors and improves the detection accuracy compared to building a single anomaly detector using the complete dataset.

The remainder of this paper is organized as follows.  Section 2 briefly introduces the related work of other researchers. We detail the proposed hybrid model in Section 3.  Section 4 provides the performance evaluation and discussion, and we summarize the paper in Section 5.

## 2. Related Work

Anomaly detection is widely used in many fields, and various methods have been proposed in past years. We refer the readers to good survey papers [3, 19–21] for more details. In this section, we review several of the most widely used anomaly detection methods and recent developments.

One-class support vector machine (OCSVM) [22] was developed from the theory of SVM to identify anomalies in the feature space by finding a hyperplane that best separates the data from the origin. Support vector data description (SVDD) [23] was also developed from SVM. Instead of finding a hyperplane, SVDD attempts to find the smallest possible hypersphere that encloses the majority of the training set while excluding potential anomalous points. Reference [24] indicated that the performance of SVM was limited on high-dimensional records due to the curse of dimensionality. In addition, OCSVM and SVDD cannot control the false alarm rate by picking hyperparameters when only normal samples are available in the training set [18].

Reference [25] proposed the local outlier factor (LOF) score to measure the degree of abnormality. The authors first found the smallest hypersphere centered at the given samples that contained the *K*-nearest neighbors. The LOF was calculated by dividing *K* by the volume of the hypersphere. Anomalous samples are usually located in a sparse region compared to normal samples. Hence, anomalous samples receive higher LOF scores. The desired decision boundary can be obtained by varying the LOF threshold. Reference [26] proposed a kNN-CF imputation method that uses the certainty factor (CF) associated with the Euclidean distance to measure the similarity in the feature space. Reference [27] proposed a one-shell neighbors imputation method to handle the missing values in given dataset.

Reference [28] proposed an ensemble classifier that combined OCSVM and the firefly algorithm. Some base one-class classifiers were first created to form the classifier pool using different subsets of the training data. The firefly algorithm was then selected as the framework to reduce the size of the classifier pool.

Reference [29] proposed a supervised outlier detection method based on the normalized residual (NR). The NR value was chosen to identify outliers and to achieve constant false alarm rate (CFAR) control. For a query point, the NR was calculated from its nearest neighbors and normalized by the median distance of the latter. Reference [30] utilized reverse nearest neighbors, rather than nearest neighbors, to determine the outliers. Reference [31] proposed the local projection score (LPS) to represent the degree of deviation of an observation relative to its neighbors. The nearest neighbors were first obtained for a given observation; then, the low-rank approximation, calculated from the nearest neighbors, was used to calculate the LPS. Observations with higher LPS were considered to be points with a high probability of being outliers. The suitable LPS threshold was difficult to determine without information about anomalous observations.

Reference [32] proposed a nonparametric method to estimate the outlier degree for each test sample. Samples with higher scores were considered likely to be outliers. They proposed a novel neighbor concept called natural neighbor (NN). Subjects A and B were NNs if A was one of the nearest neighbors of B and B was one of the nearest neighbors of A. The natural outlier factor (NOF) was calculated from the natural value to measure the outliers, but a suitable threshold for outlier degree was difficult to determine without known countersamples.

## 3. Methods

### 3.1. Deep Autoencoder

**Figure 1:**
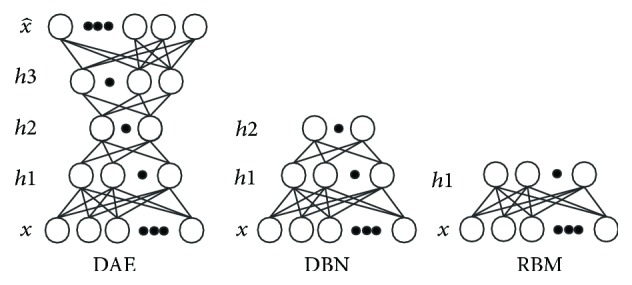
Model architecture of DAE, DBN, and RBM.

DAE was developed from DBN, which was first proposed in [15]. A DAE is composed of two symmetrical DBNs that typically have more than one shallow layer representing the encoding half of the net and corresponding to the decoding net. A DBN can be obtained by stacking multiple RBMs. An RBM is an undirected graphical model with visible units *v* representing observations and hidden units *h* learning to represent features. In contrast to the general Boltzmann machine, the nodes of an RBM are not connected at the same level. The trained RBM maps the input vector **x** (also known as *v*) to a feature space of dimensions *d* = |*h*|, where *d* < *n* and *n* is the dimensionality of *v*.  Figure 1 illustrates the model architectures of DAE, DBN, and RBM.

As an autoencoder method, DAE seeks the solution by minimizing the reconstruction error. In the simplest case, where there is one hidden layer, the DAE encoder stage maps input into a smaller feature space that can be formulated as

(1) $$\begin{array}{c} h=\sigma \left(\;W\cdot x+b\;\right), \end{array}$$

where **x** represents the input vector, *σ* is an elementwise activation function, such as a sigmoid function or a rectified linear unit, *W* is a weight matrix, and **b** is a bias vector.

In the decoder stage, the output $\hat{x}$ is reconstructed from the mapped **h**, which has the same dimensions as **x**:

(2) $$\begin{array}{c} \hat{x}=W^{\prime }\cdot h+b^{\prime }, \end{array}$$

where *W*′ is the decoding matrix and *b*′ is a vector of biases of the output layer.

The parameters are determined by optimizing the reconstruction error, such as the squared error:

(3) $$\begin{array}{c} L\left(\;x,\hat{x}\;\right)={\left\|\;x-\hat{x}\;\right\|}^{2}. \end{array}$$

In practice, the deep architecture of DBN demonstrates great power in nonlinear mapping. However, the presence of many layers implies a large number of parameters to learn, and the traditional back-propagation (BP) is not efficient without a good initialization of the weights. Thus, pretraining is adopted to improve the initialization of the parameters. One widely used pretraining method is to train each DBN layer as an individual RBM, where the hidden output of the previous layer is treated as the visible input for the subsequent layer.

RBM encodes the energy between visible input vector **v** and hidden output **h** as given by

(4) $$\begin{array}{c} E\left(\;v,h\;\right)=-\overset{M}{\underset{i=1}{\sum }}b_{i}v_{i}-\overset{N}{\underset{j=1}{\sum }}c_{j}h_{j}-\underset{i,j}{\sum }v_{i}h_{j}w_{ij}, \end{array}$$

where *v*_*i*_ and *h*_*j*_ are the visible and hidden units, respectively; *w*_*ij*_ is the weight connecting units *i* and *j*; *M* denotes the number of visible units; *N* represents the number of hidden units; and *b*_*i*_ and *c*_*j*_ are the biases for the visible and hidden units, respectively. The conditional distribution *p*(**h**∣**v**) can be calculated as

(5) $$\begin{array}{c} p\left(\;h\;|\;v\;\right)=\underset{j}{\prod }p\left(\;h_{j}\;|\;v\;\right)=\underset{j}{\prod }\sigma \left(\;\overset{N}{\underset{j=1}{\sum }}w_{ij}v_{j}+c_{j}\;\right). \end{array}$$

The conditional distribution *p*(**h**∣**v**) is calculated as

(6) $$\begin{array}{c} p\left(\;v\;|\;h\;\right)=\underset{i}{\prod }p\left(\;v_{i}\;|\;h\;\right)=\underset{i}{\prod }\sigma \left(\;\overset{M}{\underset{i=1}{\sum }}w_{ij}h_{i}+b_{i}\;\right). \end{array}$$

RBM is trained to determine the values of parameters *θ* such that (4) is minimized. After the RBM pretraining is complete, the parameters learnt on the layerwise basis are used as the initial parameters to train the whole DAE via the traditional BP algorithm.

### 3.2. Anomaly Detector

Let *S* = {*x*_1_,…, *x*_*n*_} be the given normal training set sampled from a density *f*_0_ and *x*_*i*_ ∈ *R*^*d*^. Assume that the test sample is from a mixed distribution of *f*_0_ and *f*_1_. The task of anomaly detection is to determine whether the test sample is consistent with normal data or deviates from normal under the specified significance level: *P*(declare*H*1∣*H*_*o*_) ≤ *α*. Reference [18] proves that anomaly detection is equivalent to the thresholding *p* value for multivariate data. The *p* value of a test sample *η* is defined as

(7) $$\begin{array}{c} p\left(\;\eta \;\right)=P_{0}\left(\;x:\frac{f_{1}\left(x\right)}{f_{0}\left(x\right)}\geq \frac{f_{1}\left(\eta \right)}{f_{0}\left(\eta \right)}\;\right). \end{array}$$

Equation (7) can be considered to be a mapping *η* → [0,1]. For a given significance level *α*, *η* will be declared as anomalous if *p*(*η*) ≤ *α*.

Reference [18] proposed a method to estimate the *p* value for test samples based on nearest neighborhood graphs. The *p* value was calculated from the whole dataset; thus, the computational cost increased as a quadratic function of the number of records. Reference [33] proved the effectiveness and efficiency of subsampling in anomaly detection. We propose an ensemble method to calculate the nearest neighborhood distance matrix for a test sample. This subsampling method can reduce the variance of the *k*-nearest neighbor distance and increase the robustness.

We first randomly sample *L* subsets with replacement from the entire training set. Each subset has *Q* elements denoted as *S*^*l*^ = {*x*_1_^*l*^,…, *x*_*Q*_^*l*^}, *l* = 1,…, *L*. For each element *x*_*q*_^*l*^, we calculate the *k*th nearest neighbor distance among all subsets *S*^*l*^; thus, *x*_*q*_^*l*^ has *L*  *k*th nearest neighbor distances. The real *k*th nearest neighbor distance for each element is averaged by these *L* values, which is formulated as

(8) $$\begin{array}{c} G\left(\;x_{q}^{l}\;\right)=\frac{1}{L}\overset{L}{\underset{i=1}{\sum }}\left(\;D^{i}\left(\;x_{q}^{l}\;\right)\;\right), \end{array}$$

where *D*^*i*^(·) denotes the *k*th nearest neighbor distance calculated from subset *S*^*i*^ and *G*(·) is the real *k*th nearest neighborhood distance.

The real *k*th nearest neighbor distance for a test sample *η* is calculated using the methods mentioned above; then, its estimated *p* value is calculated following (9) [18], by plugging the value into each subset *S*^*l*^:

(9) $$\begin{array}{c} \hat{p}\left(\;\eta^{l}\;\right)=\frac{1}{Q}\overset{Q}{\underset{q=1}{\sum }}I_{G\left(\eta^{l}\right)\leq G\left(x_{q}^{l}\right)}, \end{array}$$

where *𝕀* is an indicator function.

For a given false alarm rate *α*, the final decision of *η* is determined by (11):

(10) Cηl=−1,p^ηl>α1,p^ηl≤α,

(11) Cη=−1,1L∑l=1LCηl<01,1L∑l=1LCηl≥0,

where 1 denotes an anomalous sample and −1 represents a normal sample. *C*(·) represents the prediction result. We set *L* as an odd number; thus, the mean *C*(*η*^*l*^) cannot be zero. The value of *α* controls the false alarm rate in the training data.

**Figure 2:**
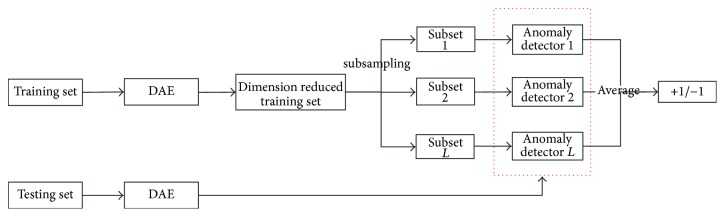
The flow chart of the proposed hybrid anomaly detection model.

The proposed model is shown in Figure 2. We also provide the persuasion of the hybrid model in Algorithm 1.

## 4. Results and Discussion

We evaluated the performance of the proposed model on several datasets and compared the proposed hybrid model with other widely used methods. Statistical tests were conducted to determine whether the differences between methods were significant [34].

### 4.1. Experimental Methodology

#### 4.1.1. Datasets and Experimental Setup

We chose four real-life datasets from the UCI Repository to form our benchmark dataset: opportunity activity recognition (OAR), gas sensor array drift (GAS), MiniBooNE particle identification dataset (MPID), and KDD 2008, with dimensionality of 110, 128, 50, and 117, respectively. Detailed information about the selected datasets is listed in Table 1.

The original OAR dataset contains 128 attributes and has four groups of labels for different tasks. In our experiment, we used only one group of labels. The original dataset was also processed by the script provided by the data owner. The dimension of the processed OAR was 110, and all records were classified into four groups representing the actions “Stand”, “Walk”, “Sit”, and “Lie”. Several experiments were conducted with this dataset. For each experiment, one class was treated as the normal class, and the others were used as the anomalous class. Similar experiments were also conducted on GAS, which contained 6 classes: “Ethanol”, “Ethylene”, “Ammonia”, “Acetaldehyde”, “Acetone”, and “Toluene”. The samples contained in MPID were labelled as “signal” and “background” and both classes were alternately used as the normal class. KDD 2008 is a breast cancer dataset that contains benign and malignant samples. There were 101617 benign records and 623 malignant records, and we used only the benign samples as the normal class. In each experiment, 80% of the normal samples were randomly selected for training, and we generated a testing set with an equal number of normal samples and anomalous samples from the remaining samples. Thus, we can evaluate both the false alarm rate and the false acceptance rate to measure the performance of the proposed model.

To better evaluate the performance of the proposed method, we compared the proposed model with other standalone algorithms, namely, SVDD, OCSVM, aK-LPE [35], and one ensemble algorithm, OCSVM with firefly (OCSVM-FA) [28]. SVDD and OCSVM were implemented in LIBSVM [36], and aK-LPE and OCSVM-FA were implemented in MATLAB following the original descriptions in [35] and [28], respectively. DAE was also implemented in MATLAB with the toolbox provided in [37].

#### 4.1.2. Performance Measurement

Various measurements have been proposed to evaluate classification performance, such as geometric means [38], *F*1 score, and recall rate. We select the area under the curve (AUC) to compare performance. The curve is the receiver operating characteristic (ROC) curve, which was developed to measure the diagnostic ability of a binary classifier system by plotting the true positive rate (TPR) against the false positive rate (FPR) at various threshold settings. The TPR and FPR are formulated as follows:

(12) FPR=FPFP+TN,TPR=TPTP+FN,

where TP denotes the number of correctly classified positive samples, TN represents the number of correctly classified negative samples, FP indicates the number of negative samples classified as positive, and FN is the number of positive samples classified as negative. In our experiments, the positive and negative samples are labelled as “+1” and “−1”, respectively. Methods with higher AUC usually perform better than those with lower AUC. Furthermore, the AUC of an ideal classifier is 1, whereas the AUC for random guessing is 0.5.

#### 4.1.3. Parameter Settings

The parameter values strongly impact the performance of anomaly detection models. Therefore, careful tuning is required to determine the most suitable parameter sets. As the determination of the best parameter set is dependent on the analyzed data, it is difficult or impossible to find a universal set of parameters that is suitable for all datasets. Therefore, we determine the optimal parameters for each dataset.

In our model, anomaly detection is performed in two stages: dimension reduction and detection. The hyperparameters of DAE, learning rate (for pretraining 0.001–0.1), number of epochs (for pretraining 5–50, for fine tuning 5–200), number of hidden units, and size of minipatch (10) are set according to [39]. If the hidden layers of the DAE are too shallow, the DAE cannot fully learn the correlations among the dimensions. However, too many hidden layers require more training records and result in greater computational cost. We empirically set 3 coding layers in our experiments. The number of subsets is also set to 3, and the *k*th nearest neighbor is set to *k* = *Q*^0.4^, following [18]. The parameters of SVDD, OCSVM, and OCSVM-FA are selected following [23], [40], and [28], respectively.

### 4.2. Results Analysis

We obtain different decision boundaries by varying *α* in our model. The different boundaries lead to different false positive and true positive rates on the test data, which form different operating points in the ROC curves used to calculate the AUC. We summarize the experimental results in Table 2. For each dataset, the algorithms were run 50 times to eliminate random effects.

Table 2 shows that our proposed method has the best AUC performance and the smallest standard deviation among the tested algorithms. The proposed model always performs better in transformed feature space than aK-LPE, which is conducted in the original high-dimensional space.

We also note that all the tested methods perform poorly on the KDD 2008 dataset when using benign records as the normal class. One possible reason is that the malignant records have a similar distribution to that of one subset of the benign records. When we build an anomaly detector from benign records, the malignant information is also treated as benign. Thus, the performance of all the methods is close to random guessing. Since only a few malignant records are provided in KDD 2008, it is challenging to train the DAE; therefore, we did not run the experiments using malignant records as the normal class.

#### 4.2.1. Statistical Analysis

In addition to obtaining the AUC values in Table 2, statistical tests were conducted to determine whether the differences among methods were significant. Both pairwise and multiple comparison tests were used. We first ran multiple comparison tests to obtain a global perspective of the performance of the algorithms over the complete dataset and then conducted pairwise tests to provide an outlook of the specific performance of the methods for a given dataset. The significance level was set to 0.1.

The Friedman rank test was used to determine whether the assigned ranks were significantly different from assigning an average rank to each classifier [41]. The Friedman test was first adopted to compare the performance of the tested anomaly detection methods and to determine their performance relationships. The Friedman rank of the AUC is shown at the bottom of Table 2, with a *p* value of 0.0002. Therefore, the null hypothesis that there is no difference between the tested methods is rejected. A smaller rank represents better performance. Furthermore, the Scheffe post hoc test was conducted for pairwise comparisons, and the results are shown in Table 3. The *p* values are all smaller than our predefined significance level of 0.1; therefore, the results are statistically significant.

#### 4.2.2. Influence of Subsets

In our experiments, we randomly generated *L* subsets to build ensemble anomaly detectors. Theoretically, the randomness in the prediction should be reduced compared to building a single classifier with the whole dataset [33]. We also determined the performance of the proposed method with varying numbers of subsets, and the results are shown in Table 4. The average AUC decreases slightly as the number of subsets increases. Meanwhile, the standard deviation of the AUC also decreases. A similar trend is observed with the other datasets. In our experiments, we set *L* to 3 as a trade-off between reduced random effects in the classification and increased computational cost. All the results reported in Table 2 were obtained with 3 anomaly detectors.

#### 4.2.3. Influence of the Nearest Neighbor

As a nonparametric method, the most important issue in KNN is to determine a suitable value of *K*. Different *K* values lead to different decision boundaries. Reference [18] found that *K* can be set to *n*^0.4^. To better evaluate the effect of *K*, we performed experiments with varying *K*. The size of the training set is set to 500, and all the results are shown in Table 5. The theoretical value of *K* is 500^0.4^ ≈ 12.

Table 5 shows that the proposed model is relatively robust to changes in *K*; that is, the performance of the proposed method is stable for a wide range of *K*. The features extracted from DAE have a more compact distribution compared with that of the original dataset, which is why our model is relatively robust to changes in *K*.

#### 4.2.4. Time Complexity

Two major factors affect the time consumption of the proposed model: DAE training and anomaly detector construction. A DAE is a parametric model that has to be trained before its first use. Training the DAE is a time-consuming task with large-scale and high-dimensional data. However, once the DAE is successfully trained, the architecture of the network is determined and the time consumed to map new test samples is negligible. Furthermore, after the original high-dimensional data are transformed into lower-dimensional space, they have a more compact distribution, and creating ensemble anomaly detectors from subsets of the dataset can greatly reduce time consumption.

In our model, the time consumed to build the anomaly detector is a quadratic function of the number of records. Assume that the time complexity to build one anomaly detector using the whole dataset is *O*(*n*^2^), where *n* is the number of records. If we created *L* anomaly detectors from *L* subsets and each subset contained 10% of the whole dataset, the time complexity would be reduced to *O*(0.01*∗L∗n*^2^).

## 5. Conclusion

In this paper, we proposed a hybrid semi-supervised anomaly detection model for high-dimensional data. The model consists of a DAE and an ensemble KNN-based anomaly detector. The DAE is trained in unsupervised mode to transform high-dimensional data into a more compact feature space. Considering that the distribution of the training set is more compact in the compact feature space, it is possible to build powerful anomaly detectors with a portion of a dataset rather than using the whole training set. The ensemble anomaly detectors have a smaller standard deviation than a single detector built from the whole dataset. Moreover, this process greatly reduces the computational cost.

The experimental results and statistical significance analysis of a wide range of real-life datasets demonstrate that the proposed model performs better than standalone algorithms. Considering that DAE training has been thoroughly researched and almost no parameters of the anomaly detector need to be trained, the hybrid model can easily be applied in various fields.

## Figures and Tables

**Algorithm 1:**
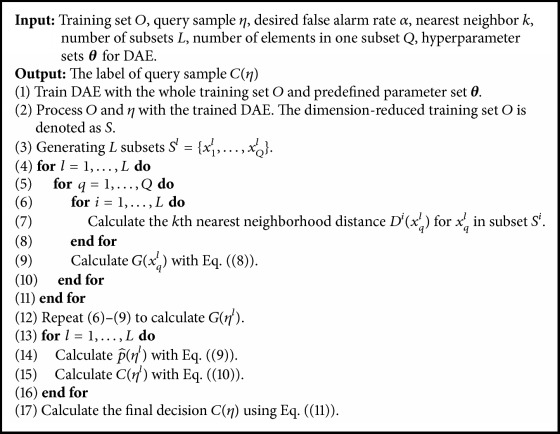
The procedure of the proposed hybrid model.

**Table 1:** Details of the datasets used in the experimental investigation.

Dataset name	Number of instances	Number of attributes	Number of classes
OAR	9699	110	4
GAS	3600	128	6
MPID	130065	50	2
KDD 2008	102240	117	2

**Table 2:** Average AUC and corresponding standard deviation of the different methods. The first class listed in the bracket indicates the normal class. Results were calculated from 50 iterations.

		SVDD	OCSVM	aK-LPE	OCSVM-FA	Our model
OAR (Stand versus others)	AUC AUC_std_	0.94 ±0.01	0.95 ±0.01	0.96 ±0.00	0.98 ±0.02	**0.99** ±0.00

OAR (Sit versus others)	AUC AUC_std_	0.88 ±0.01	0.87 ±0.02	0.90 ±0.04	0.90 ±0.01	**0.95** ±0.01

OAR (Lie versus others)	AUC AUC_std_	0.95 ±0.01	0.95 ±0.01	0.97 ±0.00	0.98 ±0.01	**0.99** ±0.00

GAS (Ethanol versus others)	AUC AUC_std_	0.92 ±0.02	0.92 ±0.02	0.94 ±0.01	0.93 ±0.02	**0.97** ±0.01

GAS (Ethylene versus others)	AUC AUC_std_	0.91 ±0.04	0.91 ±0.04	0.92 ±0.03	0.94 ±0.02	**0.98** ±0.01

GAS (Ammonia versus others)	AUC AUC_std_	0.92 ±0.02	0.93 ±0.02	0.95 ±0.01	0.96 ±0.01	**0.99** ±0.00

GAS (Acetone versus others)	AUC AUC_std_	0.81 ±0.03	0.77 ±0.04	0.80 ±0.03	0.80 ±0.03	**0.92** ±0.01

MPID (background versus signal)	AUC AUC_std_	0.70 ±0.04	0.77 ±0.03	0.75 ±0.03	0.79 ±0.03	**0.82** ±0.01

MPID (signal versus background)	AUC AUC_std_	0.66 ±0.09	0.72 ±0.11	0.73 ±0.03	0.70 ±0.03	**0.76** ±0.01

KDD 2008 (benign versus malignant)	AUC AUC_std_	0.50 ±0.03	0.51 ±0.03	0.34 ±0.01	0.50 ±0.01	**0.52** ±0.01

Rank		4.3	3.90	3.10	2.65	1.05

**Table 3:** Scheffe test for comparison of the proposed model and other methods. “+” indicates that the method on the left is better.

Methods	*p* value
Our model versus SVDD	+0.0005
Our model versus OCSVM	+0.0043
Our model versus aK-LPE	+0.0020
Our model versus OCSVM-FA	+0.0916

**Table 4:** Performance of the proposed model with different anomaly detectors.

Dataset	*L* = 1	*L* = 3	*L* = 11	*L* = 31
GAS (Acetone versus others)	0.917 ±0.022	0.916 ±0.007	0.915 ±0.007	0.914 ±0.007

OAR (Sit versus others)	0.957 ±0.017	0.951 ±0.011	0.948 ±0.008	0.941 ±0.004

**Table 5:** The average AUC of the proposed model with different *K*-nearest neighbors.

Dataset	*K* = 3	*K* = 10	*K* = 30
OAR	0.99	0.99	0.97
GAS	0.97	0.97	0.94
MPID	0.75	0.76	0.74
